# Morphology Characterization, Molecular Phylogeny, and Pathogenicity of *Diaporthe passifloricola* on *Citrus reticulata* cv. Nanfengmiju in Jiangxi Province, China

**DOI:** 10.3390/plants10020218

**Published:** 2021-01-23

**Authors:** Chingchai Chaisiri, Xiang-Yu Liu, Wei-Xiao Yin, Chao-Xi Luo, Yang Lin

**Affiliations:** 1 Hubei Key Laboratory of Plant Pathology, Huazhong Agricultural University, Wuhan 430070, China; chaisiri.ch@gmail.com (C.C.); xiangyuliu@webmail.hzau.edu.cn (X.-Y.L.); wxyin@mail.hzau.edu.cn (W.-X.Y.); cxluo@mail.hzau.edu.cn (C.-X.L.); 2 Key Lab of Horticultural Plant Biology, Ministry of Education, Huazhong Agricultural University, Wuhan 430070, China

**Keywords:** citrus, *Diaporthe passifloricola*, morphological characterization, multi-locus phylogenetic analyses

## Abstract

The Nanfengmiju (*Citrus reticulata* cv. Nanfengmiju), a high-quality local variety of mandarin, is one of the major fruit crops in Jiangxi Province, China. Citrus melanose and stem-end rot, two common fungal diseases of Nanfengmiju, are both caused by *Diaporthe* spp. (syn. *Phomopsis* spp.). Identification of the *Diaporthe* species is essential for epidemiological studies, quarantine measures, and management of diseases caused by these fungi. Melanose disease was observed on Nanfengmiju fruit in Jiangxi Province of China in 2016. Based on morphological characterization and multi-locus phylogenetic analyses, three out of 39 isolates from diseased samples were identified as *D. passifloricola*. Since these three isolates did not cause melanose on citrus fruit in the pathogenicity tests, they were presumed to be endophytic fungi present in the diseased tissues. However, our results indicate that *D. passifloricola* may persist as a symptom-less endophyte in the peel of citrus fruit, yet it may cause stem-end if it invades the stem end during fruit storage. To the best of our knowledge, this is the first report of *D. passifloricola* as the causal agent of the stem-end rot disease in *Citrus* *reticulata* cv. Nanfengmiju.

## 1. Introduction

As the earliest citrus producer in the world, China has over 4000 years of history of citrus cultivation. The citrus industry of China covers more than 20 provinces [1]. Recently, the cultivation area reached 2.5 million ha, and the production was about 38 million tons [2]. Melanose, one of the most common fungal diseases of citrus worldwide [3,4], generally occurs in many citrus-growing regions of China, such as Chongqing, Fujian, Guangdong, Guangxi, Hunan, Jiangxi, Shaanxi, Shanghai, Zhejiang, and so on [5,6,7]. All commercial citrus varieties are susceptible to melanose. Typical symptoms of melanose disease are small, discrete, sunken spots with a yellowish, reddish-brown to black color. Symptoms begin as tiny pustular lesions, then, pustular lesions disappear and become hardened gummed areas with a sandpaper-like surface [3,8,9]. *Diaporthe* spp. (syn. *Phomopsis*) are the causal agents of melanose and can also cause stem-end rots on fruit during the storage period. Since 95% of citrus is consumed as fresh fruit in China, melanose and stem-end rots diseases reduce the economic value of this crop seriously.

At present, *Diaporthe citri* is the only known causal agent of citrus melanose disease in the world. The species was first found as the causal agent of stem-end rot of citrus fruit in Florida, USA [10]. After that, *D. citri* was also associated with melanose of citrus fruit, leaves, and shoots and gummosis of perennial branches worldwide [11,12,13,14]. All *Citrus* species are susceptible to it [4]. In China, *D. citri* has been isolated in many citrus growing regions, including Guangxi [15], Guangdong [16], Fujian [17], Jiangxi [18], Sichuan [19], Taiwan, Guizhou, Yunnan, Hubei, Jiangsu [20], Zhejiang, and Shanghai [5]. In addition to *D. citri*, *D. citriasiana*, and *D. citrichinensis* have also been found to be pathogens of stem-end rot of citrus fruit in China. *D. citriasiana* distributes in Shaanxi and Jiangxi Provinces, China. *D. citrichinensis* is only found in Shaanxi Province, China [5].

The genus *Diaporthe*, belonging to the *Diaporthaceae*, Diaporthales, Ascomycota, shows high species diversity. Many species are harmful plant pathogens and exhibit broad host ranges [21,22,23,24,25]. A single species of *Diaporthe* is commonly associated with different hosts, while a single host may be infected by multiple species of *Diaporthe* [26,27]. Up to now, over 1020 names “*Diaporthe*” and around 950 names of the asexual morph “*Phomopsis*” are recorded in MycoBank lists (accessed July, 2020; http://www.mycobank.org), of which more than 100 *Diaporthe* or *Phomopsis* species have been reported in China [5,6,28,29,30,31,32,33]. In the past, morphological characteristics and host associations were the basis of the identification of *Diaporthe* species. The typical morphological characteristics of *Diaporthe* spp. are immersed ascomata and erumpent pseudostroma with elongated perithecial necks for the sexual morph [34] and black conidiomata with dimorphic conidia (alpha and beta conidia) for the asexual morph [35]. In some species, there are intermediates between alpha and beta conidia named gamma conidia [36]. However, morphological traits tend to vary in response to changes in environmental conditions, thus they may not be sufficiently reliable for the identification of *Diaporthe* at the species level [37]. With the development of molecular identification, multi-locus phylogenies combined with morphological characterization have been developed to identify *Diaporthe* species [21,24,30,37,38]. Nuclear ribosomal internal transcribed spacer regions (ITS), beta-tubulin gene (*TUB*), translation elongation factor 1-α gene (*TEF*), histone-3 gene (*HIS*), and calmodulin gene (*CAL*) are commonly employed markers to identify *Diaporthe* species [21,31,37,38].

The Nanfengmiju (*Citrus reticulata* cv. Nanfengmiju), a high-quality local variety of mandarin, is one of the major fruit crops in Jiangxi Province. The accumulation of dead citrus wood results in the increase of fungal inocula in orchards of Jiangxi. Currently, melanose has become the major fungal disease of Nanfengmiju, immensely reducing the commercial value of citrus production. The identification of *Diaporthe* spp. is essential for the epidemiology, quarantine measure, and management of citrus melanose and stem-end rot diseases. In this study, morphology, and sequences of five loci (ITS, *TUB*, *TEF*, *HIS*, and *CAL*) were employed to identify and characterize *Diaporthe* species on citrus fruit.

## 2. Results

### 2.1. Morphological Characterization of D. passifloricola

**Figure 1:**
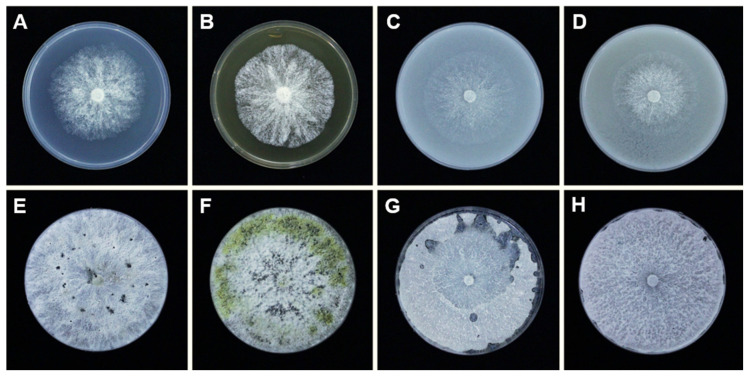
The cultural characteristics of *Diaporthe passifloricola* (NFIF-3-21) on different media. The isolate was incubated at 25 °C in the dark. (**A**,**E**), PDA medium, (**B**,**F**), MEA medium, (**C**,**G**), CMA medium, (**D**,**H**), OMA medium. Note: **A**–**D**, Colonies after 3 days incubation, **E**–**H**, Colonies after 30 days incubation.

Thirty-nine isolates (Supplementary Figure S1), were obtained from 10 diseased citrus fruit with typical melanose symptoms. Of these, three isolates preliminarily identified as *D. passifloricola* with the ITS marker were designated as NFIF-3-11, NFIF-3-19, and NFIF-3-21, and sorted out for further study. All three isolates showed the same culture characteristics on four kinds of media. After three days of incubation, the diameter of colonies on potato dextrose agar (PDA), malt extract agar (MEA), corn meal agar (CMA), and oatmeal agar (OMA) media reached 53–69 mm ($\overset{-}{x}$ = 60), 51–63 mm ($\overset{-}{x}$ = 57), 43–56 mm ($\overset{-}{x}$ = 51), and 44–51 mm ($\overset{-}{x}$ = 49), respectively. The colonies were fluffy with smooth margins. After 30 days of incubation, the surface of colonies on PDA, CMA, and OMA media had a uniform whitish appearance, whereas the colony grown on MEA presented yellowish patches (Figure 1).

**Figure 2:**
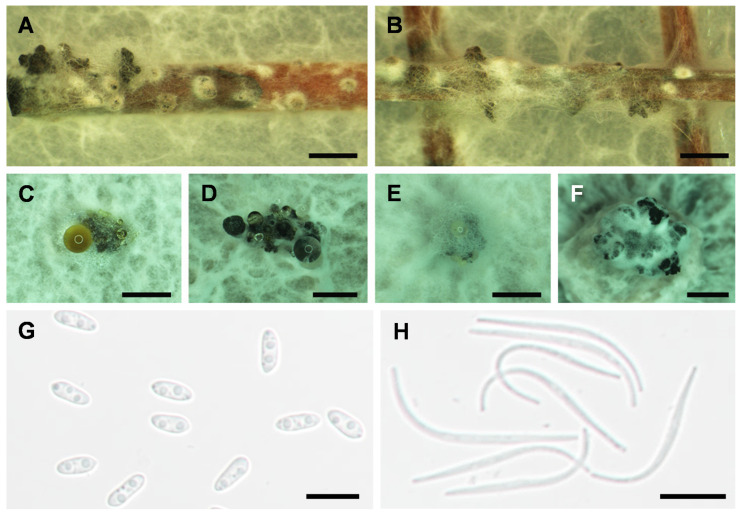
Asexual reproduction of *Diaporthe passifloricola* (NFIF-3-21). (**A**,**B)**, conidiomata on PNA after 30 days incubation, (**C**–**F**), conidiomata on PDA after 30 days incubation, (**G**), alpha (α) conidia, (**H**), beta (β) conidia. Scale bars: **A**–**B**, 500 µm; **C**–**F**, 200 µm; **G**–**H**, 10 µm.

Sporulation was induced on PDA and 1/10 PDA medium supplemented with sterilized pine needles (PNA). Conidiomata (pycnidia) were solitary to aggregated, black, sub-globose to globose, up to 200 µm in diameter. Conidial masses were hyaline to creamy, yellowish. Conidial droplets were exuded from central ostioles. Pycnidial walls consisted of 3–6 layers, medium brown (Figure 2). All three isolates produced dimorphic conidia. Alpha (α) conidia were (6.9–) 7.2–8 (–8.2) µm × 3.1–4.1 µm (*x* = 7.6 × 3.6 µm², *n* = 30), aseptate, bi-guttulate, hyaline, fusoid, and ellipsoid, smooth, apex subrounded to rounded, base subtruncate to truncate. Beta (β) conidia were (22.3–) 23.7–26.6 (–27.9) µm × 1–2 µm (*x* = 25.1 × 1.5 µm², *n* = 30), aseptate, slightly curved to spindle-shaped, smooth, base truncate. Gamma (C) conidia were not observed.

### 2.2. Pathogenicity Test

**Figure 3:**
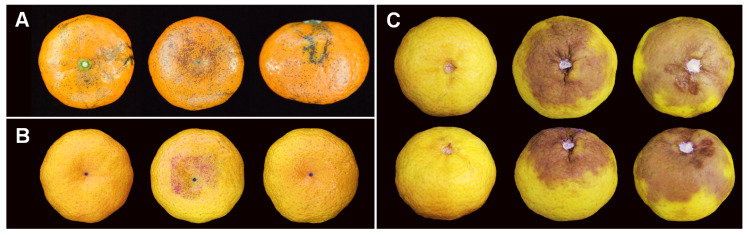
(**A**) Nanfengmiju fruit from Jiangxi Province showing symptoms of melanose. (**B**) pathogenicity stimulating melanose symptoms on mandarin fruit. For each strain, 300 μL of conidial suspensions is dropped on a piece of cotton, and then placed on the bottom of the fruit. The inoculated fruit are placed in a plastic chamber maintain 95% relative humidity, incubated at 25 °C 12 h of light and 12 h of dark for 15 days. (**C**) pathogenicity stimulating stem-end rot symptoms on stem-end of mandarin fruit. The stems of citrus fruit are removed carefully, and 10 μL of conidial suspension of each strain is dropped there and incubated at 25 °C in the dark for 7 days. Note: **B** and **C**, from left to right are sterile water, conidial suspensions of *D. citri* (isolate NFHF-8-4) and conidia suspensions of *D. passifloricola* (isolate NFIF-3-21), respectively.

In pathogenicity tests, non-wounded Nanfengmiju fruit were used to test the ability of three isolates to cause citrus melanose and stem-end rot diseases. At 15 days after inducing melanose symptom, three isolates of NFIF-3-11, NFIF-3-19, and NFIF-3-21 did not cause any symptoms, while the positive control *D. citri* strain caused typical reddish-brown to black lesion spots symptoms (Figure 3B). On the contrary, all the fruit inoculated with conidial suspension of isolates NFIF-3-11, NFIF-3-19, and NFIF-3-21, as well as positive control fruit inoculated with *D. citri* strain showed typical rot symptoms at 7 days after inoculation. No significant symptom was observed on negative control fruit inoculated with sterile water (Figure 3C). Re-isolation was performed following Koch’s postulation method. The strains were re-isolated from the experimentally inoculated fruit with stem-end rot symptoms. The identity of the re-isolated strains was confirmed by amplification and sequencing of ITS, *TUB*, *TEF*, *HIS*, and *CAL* molecular markers.

### 2.3. Phylogenetic Analyses

For preliminary identification, the MegaBlast search was performed for ITS region of three isolates in NCBI’s GenBank nucleotide database. All three isolates (NFIF-3-11, NFIF-3-19, and NFIF-3-21) showed 100% identity to *Diaporthe ueckerae* (KY565426) and *Phomopsis* sp. (KX510126, XP677503, KM229696, FJ233186, and GU595054), 99% identity to *D. phaseolorum* (LC360110), *D. longicolla* (KF577903), *D. ueckerae* (KY565424, KY565425), and *D. passifloricola* (NR_147595).

**Figure 4:**
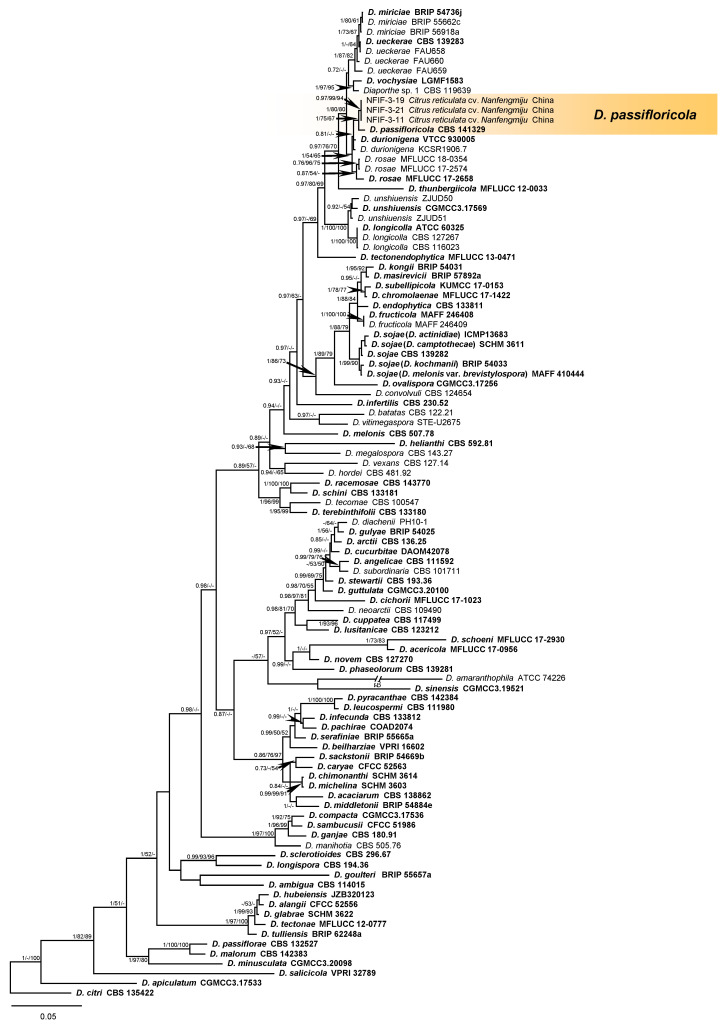
Bayesian inference phylogenetic tree is generated from the analysis of the combined sequences of five loci (ITS, *TUB*, *TEF*, *HIS*, and *CAL*). Posterior probabilities support values ≥0.7 and Bootstrap support values ≥50%, Bayesian posterior probabilities values (BIPP)/ Maximum likelihood bootstrap values (MLBS)/ Maximum parsimony bootstrap values (MPBS) are displayed at the nodes. The tree is rooted with *D. citri* CBS 135422. Ex-type, ex-epitype, and holotype cultures are indicated in **bold**. The codes of isolates used for phylogenetic tree are given.

Multi-locus phylogenetic analyses were carried out based on the sequences of ITS, *TUB*, *TEF*, *HIS*, and *CAL*. To verify if these five loci were congruent and could be combined together, single locus analysis was also performed for each locus. The results indicated that the topology of single-locus trees was congruent (Supplementary Figures S2–S6). Fifteen new sequences were generated from three isolates in this study. Other published sequences of *Diaporthe* spp. were downloaded from GenBank database. In total, 2738 characters of 101 strains from 80 *Diaporthe* spp., including one outgroup species *D. citri* (CBS 135422), were employed for Bayesian Inference (BI), Maximum Likelihood (ML), and Maximum Parsimony (MP) analyses to construct phylogenetic tree. The dataset consisted of 611 characters of ITS (1–611), 868 characters of *TUB* (612–1479), 527 characters of *TEF* (1480–2006), 581 characters of *HIS* (2007–2587), and 578 characters of *CAL* (2588–3165), respectively. MP analyses of combined data generated a single most parsimonious tree (tree length (TL) = 5416, consistency index (CI) = 0.449, retention index (RI) = 0.739, rescaled consistency index (RC) = 0.332, and homoplasy index (HI) = 0.551). Of the 3165 analyzed characters, 1036 characters were parsimony-informative, 431 variable characters were parsimony uninformative, and 1698 characters were constant. Data of each region/loci were shown in Supplementary Table S1. Using the best scoring RA×ML analysis, a final optimization tree with a likelihood value of −30,716.492582 was generated. The matrix data had 1837 distinct alignment patterns in the ML analysis, with 39.30% of gaps and completely undetermined characters. Estimated base frequencies were as follows: A = 0.212443, C = 0.325722, G = 0.238041, T = 0.223795, with substitution rates AC = 1.252910, AG = 4.007552, AT = 1.250610, C = 1.175745, CT = 5.302300, GT = 1.000000. The gamma distribution shape parameter alpha = 0.938818 and the TL = 6.170537. The ML and MP tree of combined data had similar topology to BI tree. The posterior probabilities (PP) values calculated from BI, bootstrap support (BS) values calculated from ML and MP analyses were plotted in Figure 4 and Supplementary Figure S7. The combined loci analyses grouped three isolates (NFIF-3-11, NFIF-3-19, and NFIF-3-21) together with 0.97 of Bayesian posterior probabilities values (BIPP), 99% of Maximum likelihood bootstrap values (MLBS), and 94% of Maximum parsimony bootstrap values (MPBS), respectively. The isolates were classified as *D. passifloricola* with 1 of BIPP, 75% of MLBS, and 67% of MPBS, and distinct from *D. durionigena*, *D. rosae*, *D. miriciae*, and *D. ueckerae*. The analysis of polymorphic nucleotides in each locus of *D. passifloricola*, *D. durionigene*, and *D. rosae* also found 11, 4, 4, and 11 polymorphic nucleotides in ITS, *TUB*, *TEF*, and *CAL*, respectively (Supplementary Table S2). While there was no polymorphic nucleotide in *HIS* sequence of three species.

Materials examined: CHINA, Jiangxi Province, Fuzhou city, Nanfeng district, on fruit of *Citrus reticulata* cv. Nanfengmiju, August 2016, C. Chaisiri (living culture: CCTCC M 2020452 = NFIF-3-21).

## 3. Discussion

*Diaporthe passifloricola* was identified from leaf spots on *Passiflora foetida* in Malaysia [39]. The colonies of this species on MEA, OA, and PDA are dirty white. Alpha conidia are aseptate, hyaline, smooth, guttulate, fusoid-ellipsoid, tapering towards both ends, apex subobtuse, base subtruncate, (5–) 6–7 (–9) × 2.5 (–3) µm. Gamma conidia are not observed. Beta conidia are spindle shaped, aseptate, smooth, hyaline, apex acutely rounded, base truncate, tapering from lower third towards apex, curved, (20–) 22–25 (–27) × 1.5 (–2) µm. In this study, the colonies of the isolates on PDA were dirty white, which are similar to those of *D. passifloricola* [39], *D. durionigena* [40], *D. rosae* [41], and *D. ueckerae* [42], while that of *D. miriciae* is buff [23]. Morphological characteristics of alpha (bi-guttulate) and beta conidia of three isolates are consistent with those of *D. passifloricola* ex-type strain (CBS 141329) [39]. The sizes of alpha and beta conidia of three isolates are larger than those of *D. durionigena* [40] and *D. rosae* [41]. The alpha conidia of *D. miriciae* are not described of guttulate characterized [23], and the beta conidia of *D. ueckerae* are not observed in a previous study [42]. Thus, morphological characteristics of the three isolates are the most consistent with those of *D. passifloricola*. Taking into account that morphological characteristics sometimes vary with environmental conditions, they are not always reliable to identify the isolates to species level in genus of *Diaporthe* [37]. Thus, further molecular identification is necessary.

The sequence of the ITS region was once used alone to identify *Diaporthe* species. However, there are many intraspecific variations in ITS locus of certain *Diaporthe* species. Sometimes the intraspecific variation is even greater than interspecific variation, which makes it difficult to identify *Diaporthe* species with ITS sequence alone [43,44]. Currently, multi-locus phylogenetic analyses have been applied for the identification of *Diaporthe* species [37,45]. Thus, although ITS sequences of all three isolates showed 100% similarity with *D. ueckerae* (KY565426) in this study, it was unreliable, due to many intraspecific variations in ITS regions of *Diaporthe* species.

The combined use of the five loci (i.e., ITS, *TUB*, *TEF*, *HIS*, and *CAL*) is shown to be the best way to generate a phylogenetic tree to determine the boundaries of *Diaporthe* spp. [21,31,33,37,38,45]. After preliminary identification with ITS locus, four species of *D. passifloricola*, *D. rosae*, *D. ueckerae*, and *D. miriciae* were found to have high identity to the three isolates obtained in this study. Thus, five loci of ITS, *TUB*, *TEF*, *HIS*, and *CAL* were further employed to perform phylogenetic analysis.

The main molecular traits of *D. passifloricola* have been described in 2016 [39]. For ITS region, *D. passifloricola* (KX228292.1) shows 98% (556/567) similarity to *D. miriciae* (KJ197284.1) and 90% (466/519)–93% (402/430) similarity to five *‘Phomopsis tersa’* (e.g., KF516000.1 and JQ585648.1). For *HIS* sequence, *D. passifloricola* (KX228367.1) exhibits 100% identity (380/380) to *D. absenteum* (KP293559.1) and 99% identity (378/380) to ‘*Diaporthe* sp. 1 RG-2013’ (KC343687.1). Meanwhile, for *TUB* sequence, *D. passifloricola* (MB817057) is 99% similar to ‘*Diaporthe* sp. 1 RG-2013’ (KC344171.1 (513/517)) and *D. miriciae* (KJ197264.1 (589/595)). However, the difference among *D. passifloricola* and other two species *D. durionigene* and *D.rosae*, which have the closest genetic distance with *D. passifloricola*, has not been reported. In this study, polymorphic nucleotides in ITS, *TUB*, *TEF*, and *CAL* sequences of *D. passifloricola*, *D. durionigene*, and *D. rosae* are determined and can distinguish three species well.

The taxonomy of *Diaporthe* is complex. Many *Diaporthe* spp. were classified according to different criteria, i.e., host associations, morphological characteristics [26,28,46,47], or sequences of ITS region [22,26,48]. It is suggested that only those type strains, whose identification has been widely recognized, should be accepted as references for the taxonomy of this genus [37,49,50]. Moreover, several isolates included type strains from previous publications are selected for references with phylogenetic analysis in this study. While MegaBlast search was performed for each locus on NCBI, the *Diaporthe* species showing the highest similarity with the sequencing of each locus of the isolates were not the type strains. Thus, the species identified by us are different from those retrieved by a single locus MegaBlast search on NCBI.

Before this study, 22 *Diaporthe* spp. associated with citrus were known in the world [5,6,25,37,51,52]. They are either pathogens, endophytes, or saprobes on citrus [6,11,25,52,53,54]. This is the first time that *D. passifloricola* has been isolated from *C. reticulata* cv. Nanfengmiju.

In previous studies, 15 *Diaporthe* spp. have been reported to be associated with citrus in China [5,6]. Of them, three species are pathogens on citrus, i.e., *D. citri*, *D. citriasiana*, and *D. citrichinensis*. *D. citri* is identified as the causal agents of melanose disease as well as stem-end rot disease. In addition to being a pathogen, *D. citri* is also found as an endophyte in non-symptomatic twigs and as a saprobe on dead twigs. Two species, *D. citriasiana*, and *D. citrichinensis*, can only cause stem-end rot symptom on ponkan fruit (*Citrus reticulata*) [5]. The other 12 *Diaporthe* spp. were identified as endophytes or saprobes on citrus [6]. All of these indicate that the symbiotic relationship and ecological function of *Diaporthe* spp. with citrus plants is complex and variable. 

Endophytes are defined as all organisms inhabiting plant organs which, at some time in their lives, can colonize internal plant tissues without causing significant damage to the host [55]. So defined, endophytes may also encompass asymptomatic latent pathogens. Sometimes asymptomatic fungi can cause diseases on their host plants under certain conditions. It’s reported that several *Plectosphaerella* spp. isolated from symptomless tomatoes and peppers can cause disease symptoms on tomato and pepper, and even basil and parsley when artificially inoculated [56,57]. *Epichloë festucae* is a well-known endophytic fungus of perennial ryegrass (*Lolium perenne*). However, a *E. festucae noxA* mutant is associated with severe stunting of the host as a result of hyphal hyper-branching and increased biomass [58]. Some fungal saprobes and pathogens can be isolated from rice (*Oryza sativa*) as endophytes [59]. In this study, since *D. passifloricola* isolates failed to cause melanose on citrus fruit, they are supposed to be the endophytic fungi colonizing diseased tissues with melanose symptoms. However, our results show that this species can induce stem-end rot symptoms on artificially inoculated citrus fruit. Thus, *D. passifloricola* could be a potential causal agent of stem-end rot disease during transportation and storage.

The disease spots of citrus melanose are formed by host hypersensitive response (HR). When the pathogens penetrate epidermal cells of the citrus, they are arrested and killed at the infection sites by hosts along with the development of melanose symptoms [60,61,62]. As a result, it is difficult to isolate pathogens in old disease spots. The disease spots were not newly formed, which might be the reason why we failed to isolate the pathogen causing melanose symptoms.

## 4. Materials and Methods 

### 4.1. Fungal Isolation

In 2016, 10 citrus fruit of Nanfengmiju with typical symptoms of melanose were collected from a citrus orchard in Fuzhou City of Jiangxi Province (Figure 3A). The discrete and sunken black spots were observed on the fruit surface. Pieces of small sections about 5 mm^2^ from the margin of the lesion were cut off and soaked in 75% ethanol solution for 1 min. The sections were surface disinfested with 1% sodium hypochlorite solution (NaClO) for 1 min, rinsed three times with sterilized water, dried, and then incubated on PDA plates amended with 100 μg/mL streptomycin and 100 μg/mL ampicillin at 25 °C for 2 to 5 days. Hyphal tips growing from the pieces of the sample were transferred onto fresh PDA plates and incubated at 25 °C for 30 days as previous methods [7]. After sporulation, single-spore-isolation was performed as previously described [63]. All single-spore cultures were stored on half strength PDA slants in Eppendorf tubes at 4 °C, and on dried filter paper discs at −20 °C, respectively. A living culture of *D. passifloricola* in this study was deposited in China Center for Type Culture Collection (CCTCC), Wuhan, China.

### 4.2. Morphological Characterization

Sporulation was induced on PDA, MEA, CMA, OMA, and PNA. After inoculation, isolates were incubated at 25 °C with 12 h of light and 12 h of dark for 30 days. Conidia were harvested from the top of mature pycnidia. Pycnidia were picked up from pine needles with sterile toothpicks. The length and width of 30 conidia were measured with a stage micrometer under a Motic BA200 light microscope (Motic China Group Co., Ltd., Xiamen, China). The morphology of conidiomata was observed under OLYMPUS SZX16 stereo microscope (Olympus Corporation, Tokyo, Japan). Images of conidia were captured using a digital camera Nikon Eclipse 80i on a compound light microscope (Nikon Corporation, Tokyo, Japan) imaging system. Images of culture plates were captured using Cannon 600D digital camera (Cannon Inc., Tokyo, Japan). Colony and pycnidia color was investigated with a color chart according to the method of Rayner [64].

### 4.3. Pathogenicity Test

Pathogenicity tests were carried out on detached Nanfengmiju fruit (*Citrus reticulata* cv. Nanfengmiju). Non-wounded citrus fruit were washed with tap water, then surface disinfested with 75% of ethanol and rinsed with sterile water. Pycnidia with alpha conidia were induced as mentioned above and diluted to 10^6^ conidia/mL with sterile water. To stimulate melanose symptoms, 300 μL of conidial suspensions was dropped on a piece of cotton, and then placed on the bottom of the fruit as previously described with a slight modification [65]. The inoculated fruit were placed in a plastic chamber with 95% relative humidity, incubated under the condition of 12 h of light and 12 h of dark at 25 °C for 15 days. Since *Diaporthe* spp. were the causal agents of both melanose and stem-end rot diseases on citrus fruit, their ability to cause stem-end rot symptom was also determined. The stems of citrus fruit were removed carefully, and 10 μl of conidial suspension (10^6^ conidia/mL) of each strain was inoculated onto stem ends as previously described [5]. Then, the inoculated fruit were placed in a plastic chamber with wet towel tissues at the bottom. The chamber was wrapped with plastic film to maintain 95% relative humidity and incubated at 25 °C in the dark for 7 days. In all the pathogenicity tests, the conidial suspension (10^6^ conidia/mL) of *D. citri* strain NFHF-8-4 [7] and sterile distilled water were used as positive and negative controls, respectively. Symptoms on fruit were observed. Four fruit were inoculated for each strain, and the experiments were repeated at least twice. 

To authenticate the causal agent, tissue pieces from the margin of lesions on the experimentally inoculated and diseased fruit were placed on PDA to re-isolate the fungus. Molecular identification of the isolate was performed using the sequence of ITS, *TUB*, *TEF*, *HIS*, and *CAL* loci as mentioned below.

### 4.4. DNA Extraction, PCR Amplification, and Sequencing

DNA extraction was performed as previously described [66]. Fragments of ITS, *TUB*, *TEF*, *HIS*, and *CAL* were amplified by polymerase chain reaction (PCR) using primer pairs ITS1/ITS4 [67], Bt-2a/Bt-2b [68], EF1-728F/EF1-986R [69], CYLH3F/H3-1b [68,70], and CAL-228F/CAL-737R [69], respectively. Twenty-five microliters of PCR reaction included 1 μL genomic DNA (100–500 ng/μL), 1 μL (10 mM) of each primer, 9.5 μL double-distilled water, and 12.5 μL 2× Taq PCR Master Mix (Aidlab Biotechnologies Co., Ltd., Beijing, China). PCR amplification was carried out with an initial denaturation step at 95 °C for 3 min followed by 40 cycles, consisting of a denaturation step at 95 °C for 30 sec, an annealing step for 50 sec, an elongation step at 72 °C for 2 min, and a final step at 72 °C for 5 min. The annealing temperatures were 51 °C for the amplification of partial ITS, 55 °C for the amplification of partial *TUB*, *TEF*, and *CAL*, and 58 °C for the amplification of partial *HIS*, respectively, as mentioned previously [31]. The size of PCR products was verified by gel electrophoresis in Tris-borate-EDTA (TBE) buffer using 1% agarose gel. Sequencing was carried out at Wuhan Tianyi Huiyuan Biotechnology Co., Ltd., Wuhan, China.

### 4.5. Phylogenetic Analyses

The preliminary identifications of the isolates obtained in this study were determined using newly generated ITS sequences with all available type-derived sequences listed in previous studies [6,24,25,37,51]. Based on the result of preliminary identification, *Diaporthe* species with the closest genetic distance to the isolates in this study were selected. Sequences (ITS, *TUB*, *TEF*, *HIS*, and *CAL*) of them were downloaded from NCBI’s GenBank nucleotide database (www.ncbi.nlm.nih.gov). All sequences used in this study are listed in Table 1, including 15 sequences of three new isolates. The reference isolates were selected from ex-type, ex-epitype, and holotype cultures. Five-locus phylogenetic analyses were conducted to identify isolates to species level according to previous studies [21,30,37]. Sequences of five loci (ITS, *TUB*, *TEF*, *HIS*, and *CAL*) were assembled. Alignments of assembled sequences were performed with L-INS-i iterative refinement method by MAFFT alignment, a version available online [71], and manual adjustment was conducted where it was necessary by BioEdit v.7.2.5 [72]. ML trees were generated with 1,000 replicates using RA×ML-HPC BlackBox v.8.2.10 [73], which was available on the CIPRES Science Gateway v.3.3 Web Portal [74]. The RAxML software selected general time reversible model of evolution including estimation of invariable sites (GTRGAMMA+I). MP analyses were carried out with 1,000 replicates using Phylogenetic Analyses Using Parsimony (PAUP*) v.4.0b10 [75], with tree bisection and reconnection (TBR) branch-swapping algorithm. All characters were weighted equally, and the alignment gaps were treated as missing characters. Descriptive tree statistics including TL, CI, RI, RC, and HI were calculated for parsimony analyses. MrModeltest v.2.3 [76] was used to perform statistical selection of the best-fit model of nucleotide substitution and the corrected Akaike information criterion (AIC) determined above was incorporated into evolutionary models in the analysis (Supplementary Table S1). BI analysis was performed by using MrBayes v.3.2.2, with Markov Chain Monte Carlo (MCMC) algorithm. Four simultaneous of MCMC chains were run for 20,000,000th generations, and trees were sampled frequency every 100th generations, resulting in a total of 20,000 trees, and started from a random tree topology. The calculation of BI analyses was stopped when the average standard deviation of split frequencies fell below 0.01. The first 10% of trees were discarded as burn-in phase of analysis, and the remaining 180,000 trees were summarized to calculate the PP in the majority rule consensus tree. Phylogenetic analyses and full alignment of datasets were submitted to TreeBASE (www.treebase.org) with the study ID: 27334.

## 5. Conclusions

Our results indicate that *D. passifloricola*, may occur as an asymptomatic endophyte in the peel of citrus fruit. If is manages to invade the fruit stalk, however, it may induce typical stem-end rot symptoms during transportation and storage. To the best of our knowledge, this is the first time *D. passifloricola* has been isolated from *Citrus reticulata* cv. Nanfengmiju in China and identified as a causal agent of stem-end rot disease in this crop.

## Figures and Tables

**Table 1:** GenBank accession numbers of isolates used in this study.

*Diaporthe* Species	Culture No.	Host Species	Origin	GenBank No.					Reference(s)
				ITS	*TUB*	*TEF*	*HIS*	*CAL*	
*D. acaciarum*	**CBS 138862**	*Acacia tortilis*	Tanzania	KP004460	KP004509	–	KP004504	–	[77]
*D. acericola*	**MFLUCC 17-0956**	*Acer negundo*	Italy	KY964224	KY964074	KY964180	–	KY964137	[78]
*D. alangii*	**CFCC 52556**	*Alangium kurzii*	China	MH121491	MH121573	MH121533	MH121451	MH121415	[31]
*D. amaranthophila*	ATCC 74226	*Amaranthus* sp.	USA	AF079776	–	–	–	–	[36]
*D. ambigua*	**CBS 114015**	*Pyrus communis*	South Africa	KC343010	KC343978	KC343736	KC343494	KC343252	[37]
*D. angelicae*	**CBS 111592**	*Heracleum sphondylium*	Austria	KC343027	KC343995	KC343753	KC343511	KC343269	[37]
*D. apiculatum*	**CGMCC3.17533**	*Camellia sinensis*	China	KP267896	KP293476	KP267970	–	–	[79]
*D. arctii*	CBS 136.25	*Arctium* sp.	Unknown	KC343031	KC343999	KC343757	KC343515	KC343273	[37]
*D. batatas*	CBS 122.21	*Ipomoea batatas*	USA	KC343040	KC344008	KC343766	KC343524	KC343282	[37]
*D. beilharziae*	**VPRI 16602**	*Indigofera australis*	Australia	JX862529	KF170921	JX862535	–	–	[80]
*D. caryae*	**CFCC 52563**	*Carya illinoensis*	China	MH121498	MH121580	MH121540	MH121458	MH121422	[31]
*D. chimonanthi*	**SCHM 3614**	*Chimonanthus praecox*	China	AY622993	–	–	–	–	[81]
*D. chromolaenae*	**MFLUCC 17-1422**	*Chromolaena odorata*	Thailand	MT214362	–	–	–	–	[82]
*D. cichorii*	**MFLUCC 17-1023**	*Cichorium intybus*	Italy	KY964220	KY964104	KY964176	–	KY964133	[78]
*D. citri*	**CBS 135422**	*Citrus* sp.	USA	KC843311	KC843187	KC843071	MF418281	KC843157	[25,51]
*D. compacta*	**CGMCC3.17536**	*Camellia sinensis*	China	KP267854	KP293434	KP267928	KP293508	–	[79]
*D. convolvuli*	CBS 124654	*Convolvulus arvensis*	Turkey	KC343054	KC344022	KC343780	KC343538	KC343296	[37]
*D. cucurbitae*	**DAOM 42078**	*Cucumis sativus*	Canada	KM453210	KP118848	KM453211	KM453212	–	[42]
*D. cuppatea*	**CBS 117499**	*Aspalathus linearis*	South Africa	KC343057	KC344025	KC343783	KC343541	KC343299	[37]
*D. diachenii*	PH10-1	Unknown	Lithuania	KR870866	–	–	–	–	[83]
*D. durionigena*	**VTCC 930005**	*Durio zibethinus*	Vietnam	MN453530	MT276159	MT276157	–	–	[40]
*D. durionigena*	KCSR1906.7	*Durio zibethinus*	Vietnam	MN453531	MT276160	MT276158	–	–	[40]
*D. endophytica*	**CBS 133811**	*Schinus terebinthifolius*	Brazil	KC343065	KC344033	KC343791	KC343549	KC343307	[37]
*D. fructicola*	**MAFF 246408**	*Passiflora edulis* x *P. edulis* f. flavicarpa	Japan	LC342734	LC342736	LC342735	LC342737	LC342738	[84]
*D. fructicola*	MAFF 246409	*Passiflora edulis* x *P. edulis* f. flavicarpa	Japan	LC342739	LC342741	LC342740	LC342742	LC342743	[84]
*D. ganjae*	**CBS 180.91**	*Cannabis sativa*	USA	KC343112	KC344080	KC343838	KC343596	KC343354	[37]
*D. glabrae*	**SCHM 3622**	*Bougainvillea glabra*	China	AY601918	–	–	–	–	[85]
*D. goulteri*	**BRIP 55657a**	*Helianthus annuus*	Australia	KJ197289	KJ197270	KJ197252	–	–	[23]
*D. gulyae*	**BRIP 54025**	*Helianthus annuus*	Australia	JF431299	KJ197271	JN645803	–	–	[23,86]
*D. guttulata*	**CGMCC3.20100**	Unknown	China	MT385950	MT424705	MT424685	MW022491	MW022470	[87]
*D. helianthi*	**CBS 592.81**	*Helianthus annuus*	Serbia	KC343115	KC344083	KC343841	KC343599	KC343357	[37]
*D. hordei*	CBS 481.92	*Hordeum vulgare*	Norway	KC343120	KC344088	KC343846	KC343604	KC343362	[37]
*D. hubeiensis*	**JZB320123**	*Vertis vinifera*	China	MK335809	MK500147	MK523570	–	MK500235	[88]
*D. infecunda*	**CBS 133812**	*Schinus terebinthifolius*	Brazil	KC343126	KC344094	KC343852	KC343610	KC343368	[37]
*D. infertilis*	**CBS 230.52**	*Citrus sinensis*	Suriname	KC343052	KC344020	KC343778	KC343536	KC343294	[37]
*D. kongii*	**BRIP 54031**	*Helianthus annuus*	Australia	JF431301	KJ197272	JN645797	–	–	[23,86]
*D. leucospermi*	**CBS 111980**	*Leucospermum* sp.	Australia	JN712460	–	–	–	–	[89]
*D. longicolla*	**ATCC 60325**	*Glycine max*	USA	KJ590728	KJ610883	KJ590767	KJ659188	KJ612124	[42]
*D. longicolla*	CBS 127267	*Glycine max*	Croatia	KC343199	KC344167	KC343925	KC343683	KC343441	[42]
*D. longicolla*	CBS 116023	*Glycine max*	USA	KC343198	KC344166	KC343924	KC343682	KC343440	[42]
*D. longispora*	**CBS 194.36**	*Ribes* sp.	Canada	KC343135	KC344103	KC343861	KC343619	KC343377	[37]
*D. lusitanicae*	**CBS 123212**	*Foeniculum vulgare*	Portugal	KC343136	KC344104	KC343862	KC343620	KC343378	[37]
*D. malorum*	**CBS 142383**	*Malus domestica*	Portugal	KY435638	KY435668	KY435627	KY435648	KY435658	[90]
*D. manihotia*	CBS 505.76	*Manihot utilissima*	Rwanda	KC343138	KC344106	KC343864	KC343622	KC343380	[37]
*D. masirevicii*	**BRIP 57892a**	*Helianthus annuus*	Australia	KJ197277	KJ197257	KJ197239	–	–	[23]
*D. megalospora*	CBS 143.27	*Sambucus canadensis*	Unknown	KC343140	KC344108	KC343866	KC343624	KC343382	[37]
*D. melonis*	**CBS 507.78**	*Cucumis melo*	USA	KC343142	KC344110	KC343868	KC343626	KC343384	[37]
*D. michelina*	**SCHM 3603**	*Michelia alba*	China	AY620820	–	–	–	–	[30]
*D. middletonii*	**BRIP 54884e**	*Rapistrum rugostrum*	Australia	KJ197286	KJ197266	KJ197248	–	–	[23]
*D. minusculata*	**CGMCC3.20098**	Unknown	China	MT385957	MT424712	MT424692	MW022499	MW022475	[87]
*D. miriciae*	**BRIP 54736j**	*Helianthus annuus*	Australia	KJ197282	KJ197262	KJ197244	–	–	[23]
*D. miriciae*	BRIP 55662c	*Glycine max*	Australia	KJ197283	KJ197263	KJ197245	–	–	[23]
*D. miriciae*	BRIP 56918a	*Vigna radiata*	Australia	KJ197284	KJ197264	KJ197246	–	–	[23]
*D. neoarctii*	**CBS 109490**	*Ambrosia trifida*	USA	KC343145	KC344113	KC343871	KC343629	KC343387	[37]
*D. novem*	**CBS 127270**	*Glycine max*	Croatia	KC343156	KC344124	KC343882	KC343640	KC343398	[37]
*D. ovalispora*	**CGMCC3.17256**	*Citrus limon*	China	KJ490628	KJ490449	KJ490507	KJ490570	–	[6]
*D. pachirae*	**COAD2074**	*Pachira glabra*	Brazil	MG559537	MG559541	MG559539	–	MG559535	[91]
*D. passiflorae*	**CBS 132527**	*Passiflora edulis*	South America	JX069860	KY435674	KY435633	KY435654	KY435664	[92]
*D. passifloricola*	**CBS 141329**	*Passiflora foetida*	Malaysia	KX228292	KX228387	–	KX228367	–	[39]
*D. passifloricola*	*NFIF-3-11*	*Citrus reticulata* cv. Nanfengmiju	China	*MG786598*	*MG925398*	*MG925401*	*MK238998*	*MK238995*	*This study*
*D. passifloricola*	*NFIF-3-19*	*Citrus reticulata* cv. Nanfengmiju	China	*MG786599*	*MG925399*	*MG925402*	*MK238999*	*MK238996*	*This study*
*D. passifloricola*	*NFIF-3-21*	*Citrus reticulata* cv. Nanfengmiju	China	*MG786600*	*MG925400*	*MG925403*	*MK239000*	*MK238997*	*This study*
*D. phaseolorum*	**CBS 139281**	*Phaseolus vulgaris*	USA	KJ590738	KJ610893	KJ590739	KJ659220	KJ612135	[42]
*D. pyracanthae*	**CBS 142384**	*Pyracantha coccinea*	Portugal	KY435635	KY435666	KY435625	KY435645	KY435656	[90]
*D. racemosae*	**CBS 143770**	*Euclea racemosa*	South Africa	MG600223	MG600227	MG600225	MG600221	MG600219	[93]
*D. rosae*	**MFLUCC 17-2658**	*Rosa* sp.	Thailand	MG828894	MG843878	–	–	MG829273	[41]
*D. rosae*	MFLUCC 18-0354	*Magnolia champaca*	Thailand	MG906792	MG968951	MG968953	–	–	[94]
*D. rosae*	MFLUCC 17-2574	*Senna siamea*	Thailand	MG906793	MG968952	MG968954	–	–	[94]
*D. sackstonii*	**BRIP 54669b**	*Helianthus annuus*	Australia	KJ197287	KJ197267	KJ197249	–	–	[23]
*D. salicicola*	**VPRI 32789**	*Salix purpurea*	Australia	JX862531	KF170923	JX862537	–	–	[80]
*D. sambucusii*	**CFCC 51986**	*Sambucus williamsii*	China	KY852495	KY852511	KY852507	KY852503	KY852499	[95]
*D. schini*	**CBS 133181**	*Schinus terebinthifolius*	Brazil	KC343191	KC344159	KC343917	KC343675	KC343433	[37]
*D. schoeni*	**MFLUCC 17-2930**	*Schoenus nigricans*	Italy	KY964226	KY964109	KY964182	–	KY964139	[78]
*D. sclerotioides*	**CBS 296.67**	*Cucumis sativus*	Netherlands	KC343193	KC344161	KC343919	KC343677	KC343435	[37]
*D. serafiniae*	**BRIP 55665a**	*Helianthus annuus*	Australia	KJ197274	KJ197254	KJ197236	–	–	[23]
*D. sinensis*	**CGMCC3.19521**	*Amaranthus* sp.	China	MK637451	MK660447	MK660449	MK660451	–	[96]
*D. sojae*	**CBS 139282**	*Glycine max*	USA	KJ590719	KJ610875	KJ590762	KJ659208	KJ612116	[42]
*D. sojae (D. actinidiae)*	**ICMP13683**	*Actinidia deliciosa*	New Zealand	KC145886	–	KC145941	–	–	[97]
*D. sojae (D. camptothecae)*	**SCHM 3611**	*Camptotheca acuminate*	China	AY622996	–	–	–	–	[81]
*D. sojae (D. kochmanii)*	**BRIP 54033**	*Helianthus annuus*	Australia	JF431295	–	JN645809	–	–	[42,86]
*D. sojae (D. melonis* var. *brevistylospora)*	**MAFF 410444**	*Cucumis melo*	Japan	KJ590714	KJ610870	KJ590757	KJ659203	KJ612111	[42]
*D. stewartii*	**CBS 193.36**	*Cosmos bipinnatus*	Unknown	FJ889448	JX275421	GQ250324	–	JX197415	[44,45]
*D. subellipicola*	**KUMCC 17-0153**	Unknown	China	MG746632	MG746634	MG746633	–	–	[98]
*D. subordinaria*	CBS 101711	*Plantago lanceolata*	New Zealand	KC343213	KC344181	KC343939	KC343697	KC343455	[37]
*D. tecomae*	CBS 100547	*Tabebuia* sp.	Brazil	KC343215	KC344183	KC343941	KC343699	KC343457	[37]
*D. tectonae*	**MFLUCC 12-0777**	*Tectona grandis*	Thailand	KU712430	KU743977	KU749359	–	KU749345	[99]
*D. tectonendophytica*	**MFLUCC 13-0471**	*Tectona grandis*	Thailand	KU712439	KU743986	KU749367	–	KU749354	[99]
*D. terebinthifolii*	**CBS 133180**	*Schinus terebinthifolius*	Brazil	KC343216	KC344184	KC343942	KC343700	KC343458	[37]
*D. thunbergiicola*	**MFLUCC 12-0033**	*Thunbergia laurifolia*	Thailand	KP715097	–	KP715098	–	–	[100]
*D. tulliensis*	**BRIP 62248a**	*Theobroma cacao*	Australia	KR936130	KR936132	KR936133	–	–	[101]
*D. ueckerae*	**CBS 139283**	*Cucumis melo*	USA	KJ590726	KJ610881	KJ590747	KJ659215	KJ612122	[42]
*D. ueckerae*	FAU659	*Cucumis melo*	USA	KJ590724	KJ610879	KJ590745	KJ659213	KJ612120	[42]
*D. ueckerae*	FAU658	*Cucumis melo*	USA	KJ590725	KJ610880	KJ590746	KJ659214	KJ612119	[42]
*D. ueckerae*	FAU660	*Cucumis melo*	USA	KJ590723	KJ610878	KJ590744	KJ659212	KJ612121	[42]
*D. unshiuensis*	**CGMCC3.17569**	*Citrus unshiu*	China	KJ490587	KJ490408	KJ490466	KJ490529	–	[6]
*D. unshiuensis*	ZJUD51	*Fortunella margarita* (Lour.) Swingle	China	KJ490586	KJ490407	KJ490465	KJ490528	–	[6]
*D. unshiuensis*	ZJUD50	*Fortunella margarita* (Lour.) Swingle	China	KJ490585	KJ490406	KJ490464	KJ490527	–	[6]
*D. vexans*	CBS 127.14	*Solanum melongena*	USA	KC343229	KC344197	KC343955	KC343713	KC343471	[37]
*D. vitimegaspora*	STE-U2675	*Vitis vinifera*	Taiwan	AF230749	–	–	–	–	[26]
*D. vochysiae*	**LGMF1583**	*Vochysia divergens*	Brazil	MG976391	MK007527	MK007526	MK033323	MK007528	[102]
*Diaporthe* sp. 1	CBS 119639	Man, abscess	Germany	KC343202	KC344170	KC343928	KC343686	KC343444	[37]
*Diaporthella corylina*	**CBS 121124**	*Corylus* sp.	China	KC343004	KC343972	KC343730	KC343488	KC343246	[37]

^a^ ATCC: American Type Culture Collection, Manassas, Virginia, USA; BRIP: Plant Pathology Herbarium, Department of Employment, Economic, Development and Innovation, Queensland, Australia; CBS: Westerdijk Fungal Biodiversity Institute, Utrecht, The Netherlands; CFCC: China Forestry Culture Collection Center, Beijing, China; CGMCC: China General Microbiological Culture Collection, Beijing, China; COAD: Coleção Octávio Almeida Drummond, Universidade Ferderal de Viçosa, Viçosa, Brazil; DAOM: Plant Research Institute, Department of Agriculture (Mycology), Ottawa, Canada; FAU: Isolates in culture collection of Systematic Mycology and Microbiology Laboratory, USDA-ARS, Beltsville, Maryland, USA; ICMP: International Collection of Micro-organisms from Plants, Landcare Research, Auckland, New Zealand; JZB: Culture collection of Institute of Plant and Environment Protection, Beijing Academy of Agriculture and Forestry Sciences, Beijing, China; KCSR, VTCC: Vietnam Type Culture Collection, Institute of Microbiology and Biotechnology (IMBT), Vietnam National University, Hanoi, Vietnam; HUMCC: Kunming Institute of Botany Culture Collection, Yunnan, China; LGMF: Culture collection of Laboratory of Genetics of Microorganisms, Federal University of Parana, Curitiba, Brazil; MAFF: Ministry of Agriculture, Forestry and Fisheries, Tsukuba, Ibaraki, Japan; MFLUCC: Mae Fah Luang University Culture Collection, Chiang Rai, Thailand; SCHM: Mycological Herbarium of South China Agricultural University, Guangzhou, China; STE-U: Culture collection of the Department of Plant Pathology, University of Stellenbosch, South Africa; VPRI: Victorian Plant Pathogen Herbarium, Bundoora, Australia; ZJUD: *Diaporthe* species culture collection at the Institute of Biotechnology, Zhejiang University, Hangzhou, China; Ex-type, ex-epitype, and holotype cultures are indicated in **bold**. Isolates obtained in this study are indicated in *italics.* ^b^ ITS: Nuclear ribosomal internal transcribed spacer regions; *TUB*: Beta-tubulin gene; *TEF*: Translation elongation factor 1-α gene; *HIS*: Histone-3 gene; and *CAL*: Calmodulin gene. Sequences generated in this study are indicated in italics.

## Data Availability

Alignment data generated in the current study are available in TreeBASE (accession http://purl.org/phylo/treebase/phylows/study/TB2:S27334). All sequence data are available in NCBI GenBank following the accession numbers in the manuscript.

## Supplementary Materials

The following are available online at https://www.mdpi.com/2223-7747/10/2/218/s1, Table S1 nucleotide substitution models, MP and ML alignment properties, Table S2 Polymorphic nucleotides in ITS, *TUB*, *TEF*, and *CAL* sequences of *D. passifloricola*, *D. durionigene*, and *D. rosae*, Figure S1. The prevalence of *Diaporthe* species on citrus in Jiangxi Province, China based on phylogenetic identification. Numbers (%) indicate the number of obtained isolates of certain species and the percentage among the total 140 isolates [1]. Yellow color indicate 39 isolates of *Diaporthe* sp. were found in this study, Figure S2. The phylogenetic tree is generated from the analysis of sequences of ITS locus. **A,** Maximum likelihood and **B,** Maximum parsimony. Bootstrap support values ≥50%, (MLBS/MPBS) are displayed at the nodes. The tree is rooted with *Diaporthella corylina* CBS 121124. Ex-type, ex-epitype and ex-isotype cultures are indicated in **bold**. The codes of isolates used for phylogenetic tree are given, Figure S3. The phylogenetic tree is generated from the analysis of sequences of *TUB* locus. **A,** Maximum likelihood and **B,** Maximum parsimony. Bootstrap support values ≥50%, (MLBS/MPBS) are displayed at the nodes. The tree is rooted with *Diaporthella corylina* CBS 121124. Ex-type, ex-epitype and ex-isotype cultures are indicated in **bold**. The codes of isolates used for phylogenetic tree are given, Figure S4. The phylogenetic tree is generated from the analysis of sequences of *TEF* locus. **A,** Maximum likelihood and **B,** Maximum parsimony. Bootstrap support values ≥50%, (MLBS/MPBS) are displayed at the nodes. The tree is rooted with *Diaporthella corylina* CBS 121124. Ex-type, ex-epitype and ex-isotype cultures are indicated in **bold**. The codes of isolates used for phylogenetic tree are given, Figure S5. The phylogenetic tree is generated from the analysis of sequences of *HIS* locus. **A,** Maximum likelihood and **B,** Maximum parsimony. Bootstrap support values ≥50%, (MLBS/MPBS) are displayed at the nodes. The tree is rooted with *Diaporthella corylina* CBS 121124. Ex-type, ex-epitype and ex-isotype cultures are indicated in **bold**. The codes of isolates used for phylogenetic tree are given, Figure S6. The phylogenetic tree is generated from the analysis of sequences of *CAL* locus. **A,** Maximum likelihood and **B,** Maximum parsimony. Bootstrap support values ≥50%, (MLBS/MPBS) are displayed at the nodes. The tree is rooted with *Diaporthella corylina* CBS 121124. Ex-type, ex-epitype and ex-isotype cultures are indicated in **bold**. The codes of isolates used for phylogenetic tree are given, Figure S7. The phylogenetic tree is generated from the analysis of the combined sequences of five loci (ITS, *TUB*, *TEF*, *HIS*, and *CAL*). **A,** Maximum likelihood and **B,** Maximum parsimony, bootstrap support values ≥50%, (MLBS/MPBS) are displayed at the nodes. The tree is rooted with *D. citri* CBS 135422. Ex-type, ex-epitype and holotype cultures are indicated in **bold**. The codes of isolates used for phylogenetic tree are given.
