# Supplementary material for: Morphology Characterization, Molecular Phylogeny, and Pathogenicity of Diaporthe passifloricola on Citrus reticulata cv. Nanfengmiju in Jiangxi Province, China
Source: Plants (Basel). 2021 Jan 23;10(2):218. doi: 10.3390/plants10020218 (PMC7911537; doi:10.3390/plants10020218)
Supplement: Supplementary file 1 [file plants-10-00218-s001.pdf]

Morphology Characterization, Molecular Phylogeny, and Pathogenicity of *Diaporthe passifloricola* on *Citrus reticulata* cv. *Nanfengmiju* in Jiangxi Province, China

Chingchai Chaisiri [1,2], Xiang-Yu Liu [1,2], Wei-Xiao Yin [1], Chao-Xi Luo [1,2] and Yang Lin [1,*]

[1] Hubei Key Laboratory of Plant Pathology, Huazhong Agricultural University, Wuhan 430070, China; chaisiri.ch@gmail.com (C.C.); xiangyuliu@webmail.hzau.edu.cn (X.-Y.L.); wxyin@mail.hzau.edu.cn (W.-X.Y.); cxluo@mail.hzau.edu.cn (C.-X.L.)

[2] Key Lab of Horticultural Plant Biology, Ministry of Education, Huazhong Agricultural University, Wuhan 430070, China

* Correspondence: yanglin@mail.hzau.edu.cn (Y.L.)

Supplementary Material

Table S1. nucleotide substitution models, MP and ML alignment properties.

	Region/gene [a]		ITS	*TUB*	*TEF*	*HIS*	*CAL*
	No. of taxa analyzed		101	88	89	58	58
	AIC nucleotide substitution model		HKY+I+G	HKY+I+G	HKY+I+G	GTR+I+G	HKY+I+G
MP	Characters included (with gaps)		611	868	527	581	578
	Invariable characters		375	456	221	371	275
	Parsimony informative characters		130	286	249	147	224
	Uninformative variable characters		106	126	57	63	79
	MP Tree length		763	1,236	1428	831	813
	Consistency index (CI)		0.446	0.513	0.432	0.569	0.450
	Retention index (RI)		0.687	0.764	0.819	0.795	0.662
	Rescaled consistency index (RC)		0.306	0.392	0.354	0.453	0.298
	Homoplasy index (HI)		0.554	0.487	0.568	0.431	0.550
ML	Matrix data distinct alignment patterns		305	533	406	268	359
	Proportion of gaps and completely undetermined characters (%)		11.00	31.29	35.05	19.62	13.21
	Final ML optimization likelihood value		−4488.074972	−7609.877475	−7162.092053	−4451.580737	– 5109.256642
	ML Tree Length		7.522317	2.205523	5.012763	3.123529	1.855683
	Gamma distribution shape parameter α		0.332481	1.513512	2.776265	1.070465	2.831607
	Estimated base frequencies	: A	0.246158	0.194971	0.220438	0.185957	0.196481
		: C	0.268463	0.362216	0.324573	0.393657	0.306183
		: G	0.256610	0.227951	0.206457	0.225053	0.266316
		: T	0.228769	0.214862	0.248531	0.195334	0.231020
		: A-C	1.099693	0.844106	1.626526	1.407074	1.255762
		: A-G	5.529640	4.296600	3.886337	3.716687	4.167474
		: A-T	1.419461	0.921182	1.332342	1.708299	1.076839
		: C-G	0.955221	0.970776	1.347249	1.234273	1.143587
		: C-T	9.669130	3.773201	5.002268	6.366428	4.537714
		: G-T	1.000000	1.000000	1.000000	1.000000	1.000000

[a] ITS: nuclear ribosomal internal transcribed spacer regions; *TUB*: beta-tubulin gene; *TEF*: translation elongation factor 1-α gene; *HIS*: histone-3 gene; and *CAL*: calmodulin gene.

Table S2. Polymorphic nucleotides in ITS, *TUB*, *TEF*, and *CAL* sequences of *D. passifloricola*, *D. durionigene*, and *D. rosae*.

Species	Isolates [a]	ITS													
		13	52	91	105	106	117	119	120	121	124	125	138	203	416
D. passifloricola	**CBS 141329**	C	C	T	C	C	T	G	T	G	A	A	G	–	G
	NFIF-3-11	–	T	•	T	A	•	•	•	•	•	•	A	–	•
	NFIF-3-19	–	T	•	T	A	•	•	•	•	•	•	A	–	•

	NFIF-3-21	–	T	•	T	A	•	•	•	•	•	•	A	–	•
D. durionigene	**VTCC 930005**	–	–	C	T	A	C	C	–	–	T	G	A	–	•
	KCSR1906.7	–	–	C	T	A	C	C	–	–	T	G	A	–	•
D. rosae	**MFLUCC 17-2658**	T	T	C	•	A	C	C	–	–	T	G	A	A	A
	MFLUCC 17-2574	–	T	•	T	A	C	C	–	–	T	G	A	•	•
	MFLUCC 18-0354	–	T	•	T	A	C	C	–	–	T	G	A	•	•

Species	Isolates [a]	*TUB*				*TEF*			
		124	133	276	377	69	227	344	346
D. passifloricola	**CBS 141329**	A	A	C	C	*Sequence not available*			
	NFIF-3-11	G	G	•	T	–	G	T	C
	NFIF-3-19	G	G	•	T	A	•	•	•
	NFIF-3-21	G	G	•	T	–	•	•	•
D. durionigene	**VTCC 930005**	G	G	•	T	–	•	–	–
	KCSR1906.7	–	•	•	•	–	•	–	–
D. rosae	**MFLUCC 17-2658**	–	•	T	T	*Sequence not available*			
	MFLUCC 17-2574	–	•	T	T	–	C	C	A
	MFLUCC 18-0354	–	•	T	T	–	C	C	A

Species	Isolates [a]	*CAL*										
		67	198	205	208	217	225	251	395	443	446	503
D. passifloricola	**CBS 141329**	*Sequence not available*										
	NFIF-3-11	C	T	A	A	T	A	G	C	T	T	A
	NFIF-3-19	•	•	•	•	•	•	•	•	•	•	•
	NFIF-3-21	•	•	•	•	•	•	•	•	•	•	•
D. durionigene	**VTCC 930005**	*Sequence not available*										
	KCSR1906.7	*Sequence not available*										
D. rosae	**MFLUCC 17-2658**	T	C	C	G	C	G	A	T	C	–	G
	MFLUCC 17-2574	*Sequence not available*										
	MFLUCC 18-0354	*Sequence not available*										

[a] Isolates in this study are indicated in *italics*, Ex-type cultures are indicated in **bold**. Dashes (–) and dots (•) indicate the gaps and identical nucleotides in the sequences, respectively.

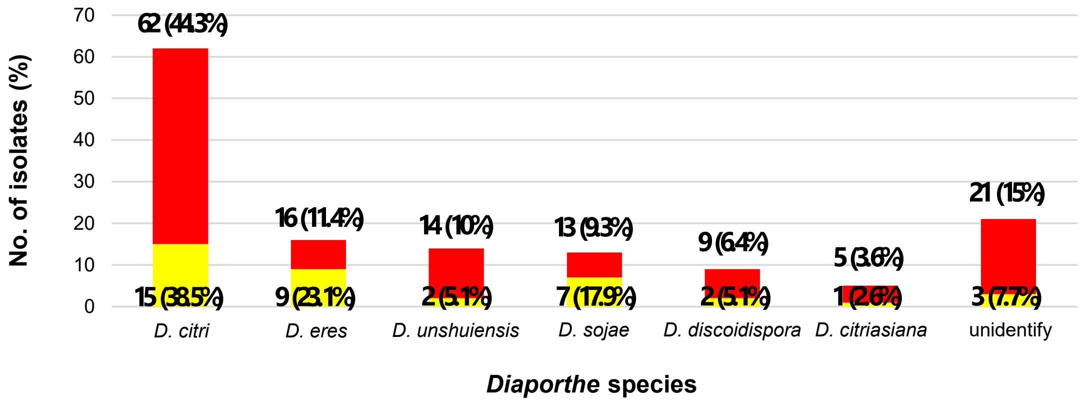

Figure S1. The prevalence of *Diaporthe* species on citrus in Jiangxi Province, China based on phylogenetic identification. Numbers (%) indicate the number of obtained isolates of certain species and the percentage among the total 140 isolates [1]. Yellow color indicate 39 isolates of *Diaporthe* sp. were found in this study.

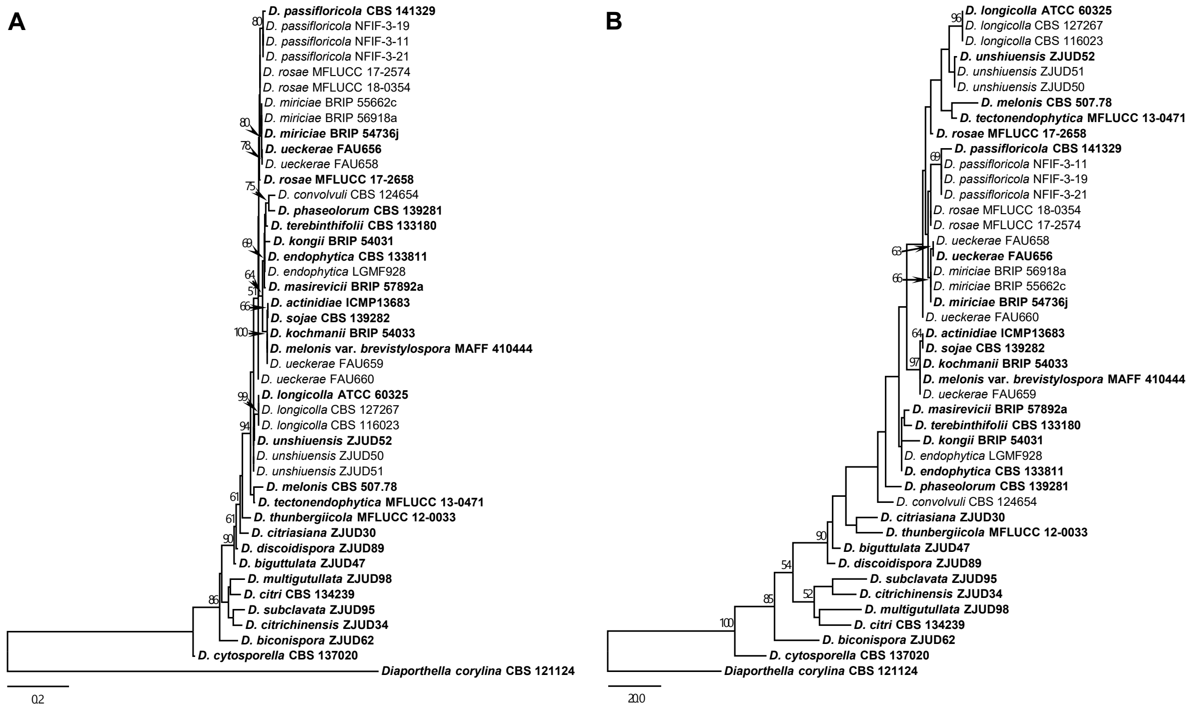

Figure S2. The phylogenetic tree is generated from the analysis of sequences of ITS locus. **A,** Maximum likelihood and **B,** Maximum parsimony. Bootstrap support values ≥50%, (MLBS/MPBS) are displayed at the nodes. The tree is rooted with *Diaporthella corylina* CBS 121124. Ex-type, ex-epitype and ex-isotype cultures are indicated in **bold**. The codes of isolates used for phylogenetic tree are given.

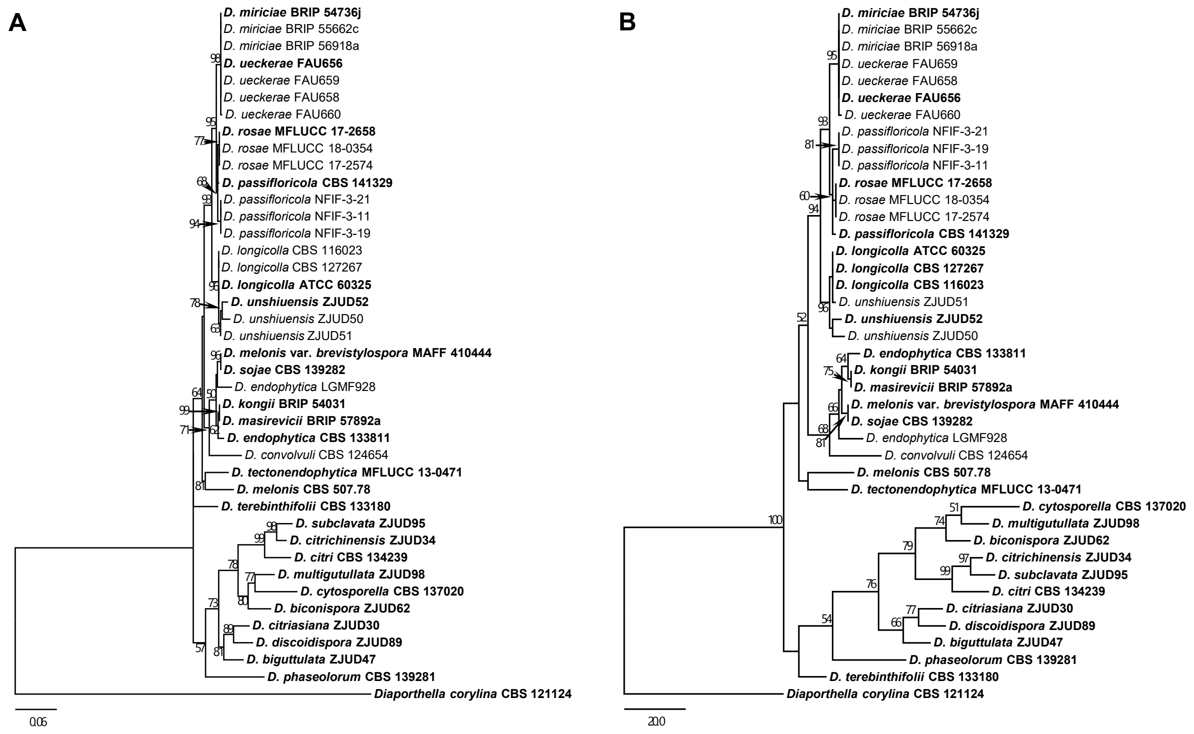

Figure S3. The phylogenetic tree is generated from the analysis of sequences of *TUB* locus. **A,** Maximum likelihood and **B,** Maximum parsimony. Bootstrap support values ≥50%, (MLBS/MPBS) are displayed at the nodes. The tree is rooted with *Diaporthella corylina* CBS 121124. Ex-type, ex-epitype and ex-isotype cultures are indicated in **bold**. The codes of isolates used for phylogenetic tree are given.

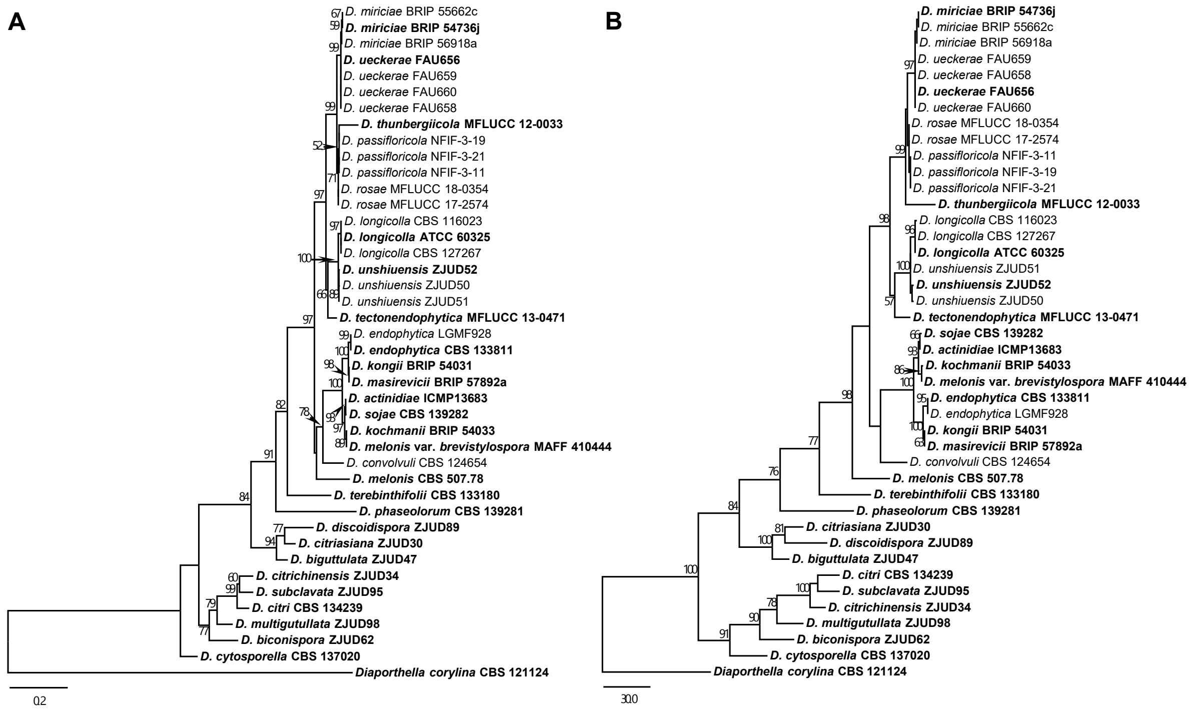

Figure S4. The phylogenetic tree is generated from the analysis of sequences of *TEF* locus. **A,** Maximum likelihood and **B,** Maximum parsimony. Bootstrap support values ≥50%, (MLBS/MPBS) are displayed at the nodes. The tree is rooted with *Diaporthella corylina* CBS 121124. Ex-type, ex-epitype and ex-isotype cultures are indicated in **bold**. The codes of isolates used for phylogenetic tree are given.

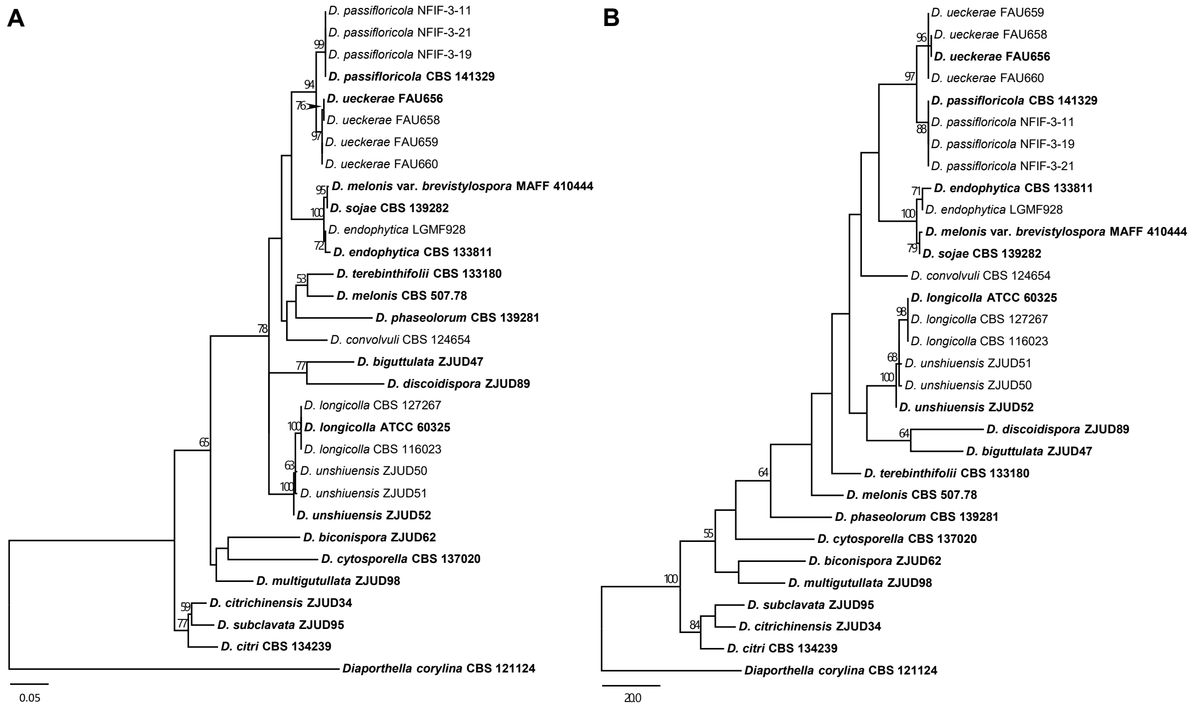

Figure S5. The phylogenetic tree is generated from the analysis of sequences of *HIS* locus. **A,** Maximum likelihood and **B,** Maximum parsimony. Bootstrap support values ≥50%, (MLBS/MPBS) are displayed at the nodes. The tree is rooted with *Diaporthella corylina* CBS 121124. Ex-type, ex-epitype and ex-isotype cultures are indicated in **bold**. The codes of isolates used for phylogenetic tree are given.

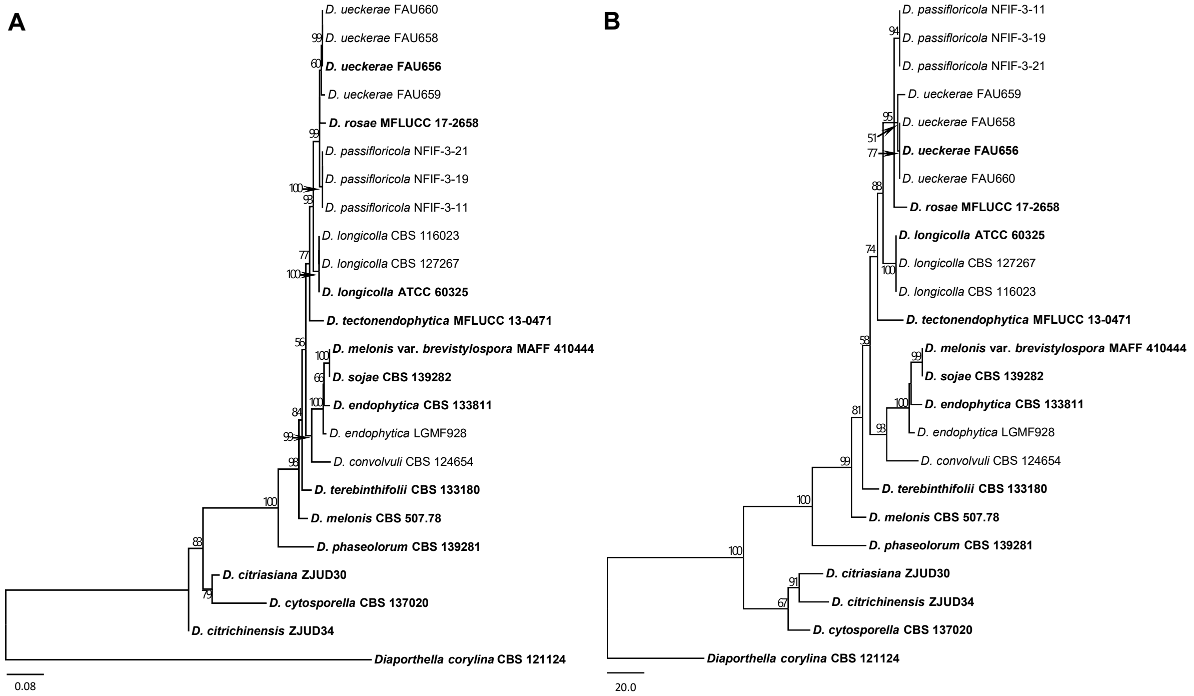

Figure S6. The phylogenetic tree is generated from the analysis of sequences of *CAL* locus. **A,** Maximum likelihood and **B,** Maximum parsimony. Bootstrap support values ≥50%, (MLBS/MPBS) are displayed at the nodes. The tree is rooted with *Diaporthella corylina* CBS 121124. Ex-type, ex-epitype and ex-isotype cultures are indicated in **bold**. The codes of isolates used for phylogenetic tree are given.

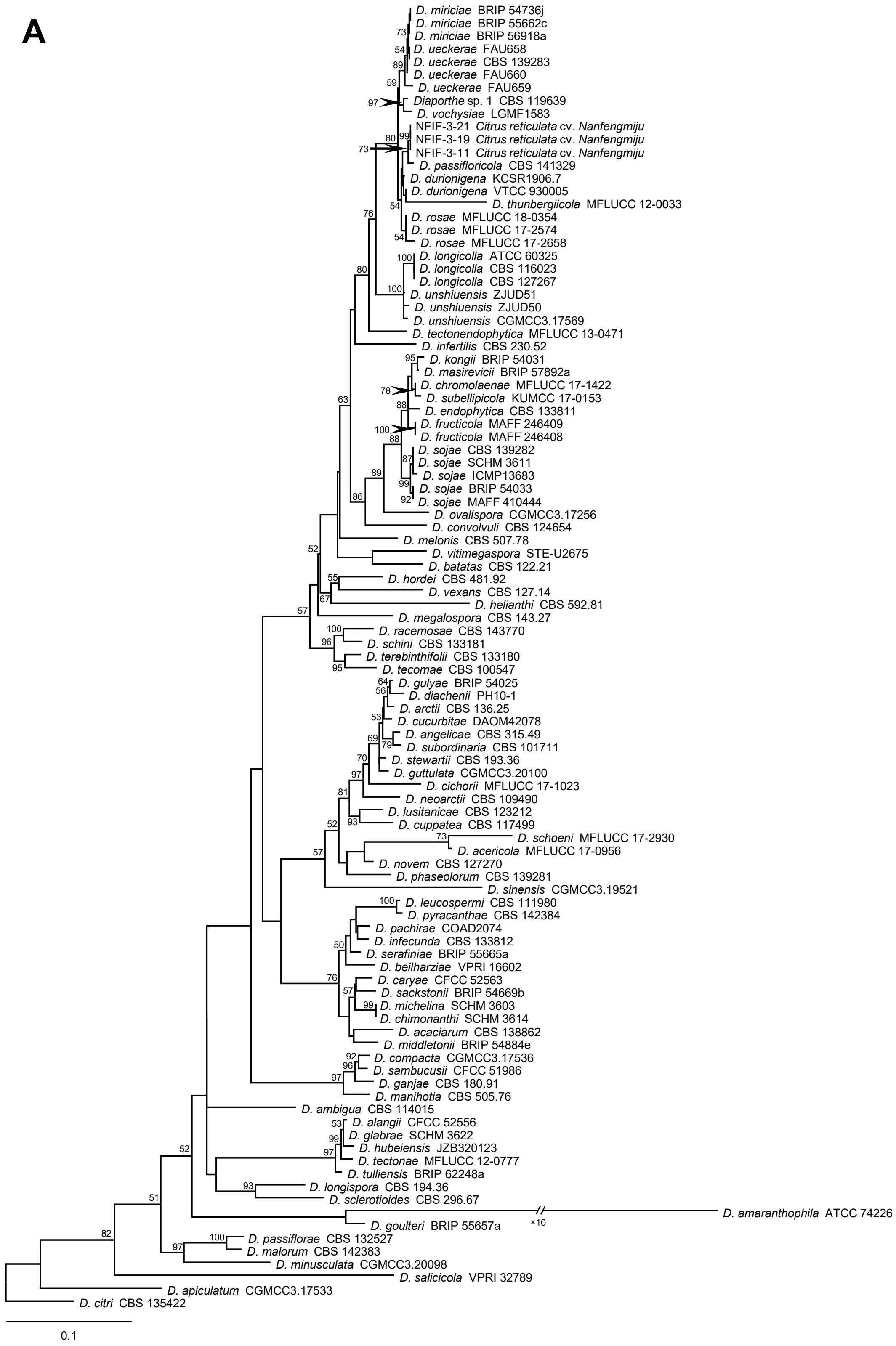

Figure S7. The phylogenetic tree is generated from the analysis of the combined sequences of five loci (ITS, *TUB*, *TEF*, *HIS*, and *CAL*). **A,** Maximum likelihood and **B,** Maximum parsimony, bootstrap support values ≥50%, (MLBS/MPBS) are displayed at the nodes. The tree is rooted with *D. citri* CBS 135422. Ex-type, ex-epitype and holotype cultures are indicated in **bold**. The codes of isolates used for phylogenetic tree are given.

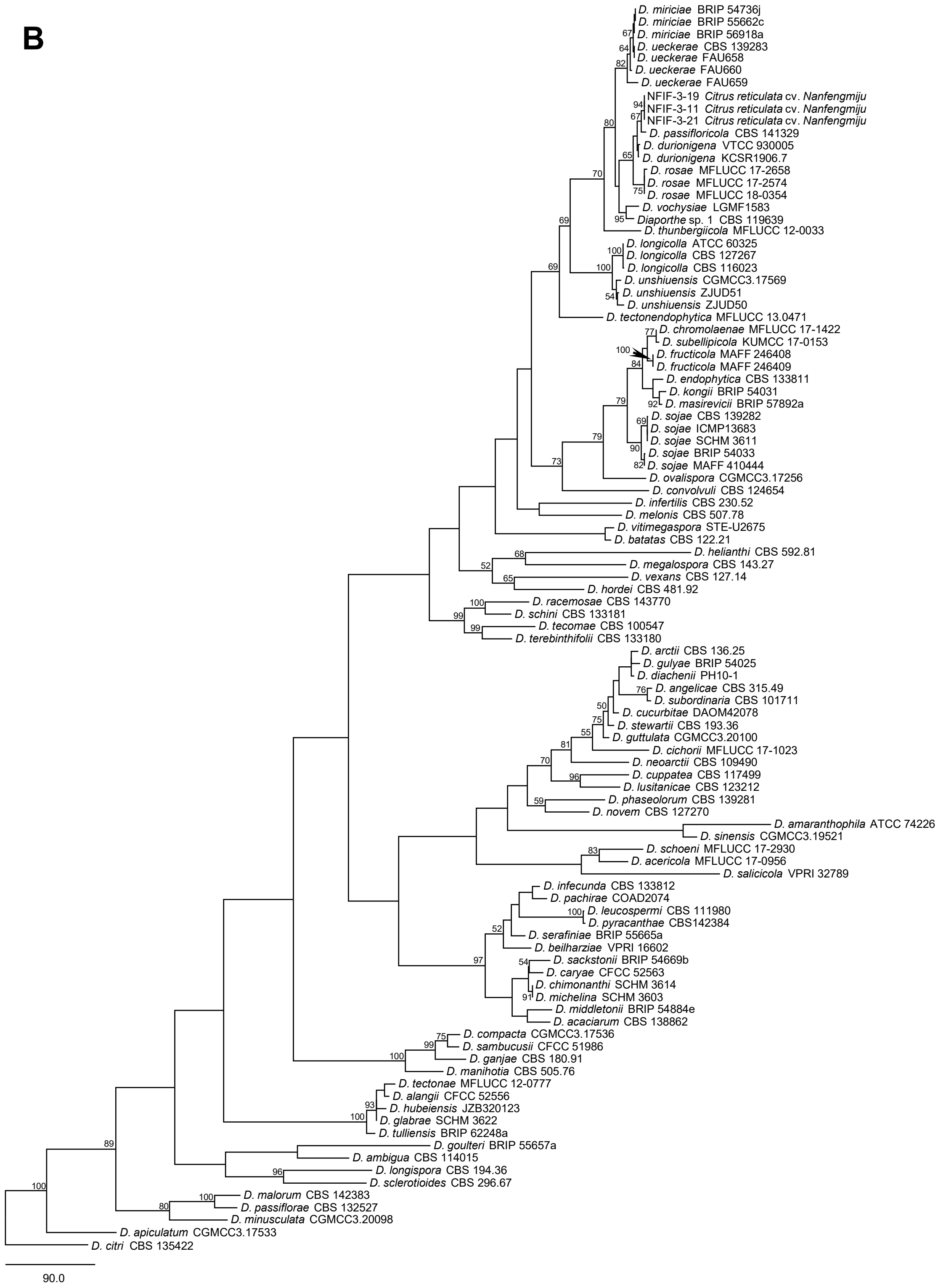

Figure S7. (Continued).

Reference:

1. Chaisiri, C.; Liu, X.Y.; Lin, Y.; Li, J.B.; Xiong, B.; Luo, C.X. Phylogenetic analysis and development of molecular tool for detection of *Diaporthe citri* causing melanose disease of citrus. *Plants* **2020**, *9*, 329.
